# Impact of *Strongyloides stercoralis* infection on complement activation in Type 2 diabetes mellitus: Insights from a clinical and anthelmintic intervention study

**DOI:** 10.1371/journal.pntd.0012048

**Published:** 2024-04-02

**Authors:** Anuradha Rajamanickam, Bindu Dasan, Saravanan Munisankar, Sujatha Nott, Pradeep A. Menon, Fayaz Ahamed Shaik, Ponnuraja Chinnaiyan, Thomas B. Nutman, Subash Babu

**Affiliations:** 1 NIH-NIAID-International Center for Excellence in Research, Chennai, India; 2 Infectious Diseases, Dignity Health, Chandler, Arizona, United States of America; 3 National Institute for Research in Tuberculosis, Chennai, India; 4 Laboratory of Parasitic Diseases, National Institute of Allergy and Infectious Diseases, National Institutes of Health, Bethesda, Maryland, United States of America; James Cook University, AUSTRALIA

## Abstract

**Background:**

Numerous studies indicate a potential protective role of helminths in diabetes mellitus (DM) progression. The complement system, vital for host defense, plays a crucial role in tissue homeostasis and immune surveillance. Dysregulated complement activation is implicated in diabetic complications. We aimed to investigate the influence of the helminth, *Strongyloides stercoralis* (*Ss*) on complement activation in individuals with type 2 DM (T2D).

**Methodology:**

We assessed circulating levels of complement proteins (C1q, C2, C3, C4, C4b, C5, C5a, and MBL (Lectin)) and their regulatory components (Factor B, Factor D, Factor H, and Factor I) in individuals with T2D with (n = 60) or without concomitant *Ss* infection (n = 58). Additionally, we evaluated the impact of anthelmintic therapy on these parameters after 6 months in *Ss*-infected individuals (n = 60).

**Results:**

*Ss*+DM+ individuals demonstrated reduced levels of complement proteins (C1q, C4b, MBL (Lectin), C3, C5a, and C3b/iC3b) and complement regulatory proteins (Factor B and Factor D) compared to *Ss*-DM+ individuals. Following anthelmintic therapy, there was a partial reversal of these levels in *Ss*+DM+ individuals.

**Conclusion:**

Our findings indicate that Ss infection reduces complement activation, potentially mitigating inflammatory processes in individuals with T2D. The study underscores the complex interplay between helminth infections, complement regulation, and diabetes mellitus, offering insights into potential therapeutic avenues.

## Author summary

Our research delves into the intricate link between *Strongyloides stercoralis*, a microscopic worm, and diabetes. Within our body, we possess a defense mechanism known as the complement system, essential for overall well-being. However, diabetes can disrupt this system. Our study aimed to uncover whether the presence of *Ss* infection influence this process. Individuals with both diabetes and *Ss* infection displayed lower levels of specific proteins associated with the complement system. Intriguingly, treating the *Ss* infection led to a partial restoration of these protein levels. This suggests that the *Ss* infection might play a role in curbing inflammation associated with diabetes. The unexpected alliance between this *Ss* infection and our body opens new avenues for potential therapies. This study highlights the complexity of interactions within our immune system and offers insights into how unanticipated allies might contribute to managing diabetes. Understanding such connections could pave the way for innovative approaches to diabetes treatment, emphasizing the importance of exploring unconventional aspects of our biological defenses.

## 1. Introduction

Numerous epidemiological studies across India, China, Indonesia, and Australia have uncovered a peculiar inverse relationship between helminth infections and diabetes mellitus (DM) [1–4]. Intriguingly, mouse models of DM have demonstrated that infection with various helminth species can restore glucose levels and enhance insulin sensitivity [5–9]. With a staggering 537 million adults (20–79 years) currently grappling with diabetes, a number projected to reach 643 million by 2030 and a daunting 783 million by 2045, understanding factors influencing DM pathophysiology is paramount [10].

The complement system and its regulatory proteins have been implicated in the complications arising from diabetes, as evidenced by extensive clinical and experimental data [11]. The complement system is a driving force in the pathogenesis of type 2 diabetes (T2D)-related issues, including metabolic dysregulation in adipose tissue, focal inflammation, endothelial dysfunction, and insulin resistance [11–13]. Recent research has shed light on the link between diabetic complications and chronic inflammation mediated by the complement system [1]. Elevated complement C3 levels have been associated with increased risks of complications such as retinopathy, nephropathy, and neuropathy [2]. In obesity-induced adipocytes, the overproduction of C3desArg (ASP) contributes to insulin resistance and chronic inflammation [3]. The continuous activation of the complement alternative pathway in T2D with obesity and dyslipidemia significantly contributes to microvascular complications [3].

While numerous studies have explored the role of complement in diabetes, the impact of *Strongyloides stercoralis* (*Ss*) infection on complement levels in individuals with DM remains uncharted territory. In this context, we hypothesize that *Ss* infections could modulate systemic complement proteins and regulatory proteins, potentially offering a preventive measure against diabetic complications. This study aims to elucidate these intricate interactions by assessing circulating levels of complement proteins and regulatory components in individuals with DM, both with and without concurrent *Ss* infection, and exploring the effects of anthelmintic therapy after six months on these parameters in *Ss*-infected individuals.

## 2. Materials and methods

### 2.1. Ethical statement

All participants were examined as part of a natural history study protocol (NCT01547884 and NCT00342576) approved by the Institutional Review Boards of the National Institute of Allergy and Infectious Diseases (USA) and the National Institute for Research in Tuberculosis (India), and informed written consent was obtained from all participants.

### 2.2. Study population

In total, we screened 2351 individuals for *Strongyloides stercoralis* (*Ss*) and Diabetes mellitus (DM). Among the screened individuals, 768 (33%) were positive for *Strongyloides stercoralis* (*Ss*) alone and 459 (20%) were diagnosed with Diabetes mellitus (DM) [4]. From this population, we recruited 118 individuals consisting of 60 clinically asymptomatic *Ss*-infected individuals with DM (hereafter *Ss*+DM+), and 58 individuals with DM and no *Ss* infection (hereafter *Ss-*DM*+*) in Kanchipuram District, Tamil Nadu, South India. This is an observational cross-sectional study. All study participants had no previous history of helminth infections or anthelmintic treatment and were HIV seronegative. All recruited individuals were confirmed negative for other viral infections, as well as active and latent tuberculosis. This is the same cohort of individuals in our previously published reports [5–8].

Blood samples were collected in EDTA, Heparin and serum tubes (BD Biosciences), and plasma or serum samples were collected through centrifugation and stored at -80°C. All methods were carried out following all the institutional committee guidelines and regulations. Overnight fasting samples were used to measure all biochemical parameters except for random blood glucose. Serum samples were used for the biochemical parameters and plasma samples for the remaining experiments.

### 2.3. Measurement of anthropometric and biochemical parameters

Anthropometric measurements, including height, weight, and waist circumference, and biochemical parameters, including random blood glucose (RBG), HbA1c, urea, creatinine, ALT, and AST were obtained using standardized techniques as detailed elsewhere [9].

### 2.4. Parasitological examination and anthelmintic treatment

*Ss* infection was diagnosed by the presence of IgG antibodies to the recombinant NIE antigen as described previously [10,11]. Further confirmation was by stool microscopy using the agar culture method [12]. Stool microscopy was also used to exclude the presence of other intestinal helminth infections. Filarial infection was excluded in all study participants by being negative in tests for circulating filarial antigen. All *Ss*+ individuals were treated with a single dose of ivermectin (12mg) and albendazole (400 mg) and follow–up blood draws were obtained six months later. We conducted comprehensive assessments including measuring IgG antibodies to the recombinant NIE antigen and confirmed the results through stool microscopy using the agar culture method. Notably, all participants tested negative for *S*. *stercoralis* at the six-month follow-up.

### 2.5. Determination of DM status

Based on American Diabetes Association criteria, diabetes was confirmed by an HbA1c value of 6.5% or greater and a random blood glucose of >200 mg/dl. Overnight fasting samples were used to measure all biochemical parameters. All diabetic individuals were newly diagnosed, not on any anti-diabetic medication at the time of the blood draw, and without any known complications or co-morbidities. All individuals were referred to the primary health care centre for diabetic treatment. The individuals who had a previous history of diabetes complications, neurological problems, and renal and cardio-related problems were excluded from the study.

### 2.6. Measurement of complement and complement regulatory proteins

Systemic plasma levels of C1q, C2, C3, C4, C4b, C5, C5a, MBL complement proteins and Factor B, Factor D, Factor H, and Factor I were determined using the Luminex xMAP technology with MILLIPLEX Bead-Based Multiplex Complement Panel I (Catalogue Number: HCMP1MAG-19K) and II (Catalogue Number: HCMP2MAG-19K) Assay kits. The lower detection limits were as follows: C2: 1.37 ng/mL; C4b: 1.37 ng/mL; C5: 2.74 ng/mL; C5a: 4.12 pg/mL; Adipsin/ Complement Factor D: 0.069 ng/mL; Mannose-Binding Lectin (MBL): 0.137 ng/mL; Complement Factor I: 0.69 ng/mL; C1q: 0.08 ng/mL; C3: 0.27 ng/mL; C3b/iC3b: 8.2 ng/mL; C4: 0.55 pg/mL; complement Factor B: 0.08 ng/mL and complement Factor H: 0.041 ng/mL.

### 2.7. Statistical analysis

Sample size calculation was done to obtain a power of 80% and an α error of 0.05%. Geometric means (GM) were used for measurements of central tendency. The *Ss+* group was compared to *Ss-* group by Mann-Whitney U tests and before and after treatment parameters were compared by Wilcoxon signed-rank test. Multiple comparisons were corrected using Holm’s correction. Analyses were performed using Graph-Pad PRISM Version 9.4.0. (GraphPad, San Diego, CA). PCA analysis was done using JMP 17 software.

## 3. Results

### 3.1. Study population characteristics

The baseline demographic characteristics and biochemical parameters have been described previously [5–8,13] and there were no significant differences in age, sex, BMI or other biochemical parameters between the 2 groups (Table 1).

**Table 1:** Demographic and Biochemical parameters.

Parameters	*Ss*+DM+	*Ss*-DM+	p-value
	n = 60	n = 58	
Gender Male/Female	30/30	30/28	NS
Age in years median (years, Range)	46 (24–63)	45 (22–63)	NS
Random Blood Glucose (mg/dl) GM (Range)	179 (140–438)	180.5 (140–198)	NS
Hemoglobin A1c (%) GM (Range)	8.57 (6.5–12.5)	8.9 (6.5–11.8)	NS
Urea (mg/dl) GM (Range)	19.5 (12.34)	21.9 (11–42)	NS
Creatinine (mg/dl) GM (Range)	0.78 (0.3–1)	0.85 (0.6–1.0)	NS
Alanine aminotransferase (U/L) GM (Range)	17.7 (7–60)	22.4 (7–92)	NS
Aspartate aminotransferase (U/L) GM (Range)	27.8 (16–110)	24.7 (11–68)	NS

GM-Geometric Mean; Hemoglobin A1c - glycated hemoglobin; NS-Not Significant

### 3.2. Diminished circulating levels of complement proteins and complement regulatory proteins in *Ss*+DM+ individuals

**Fig 1:**
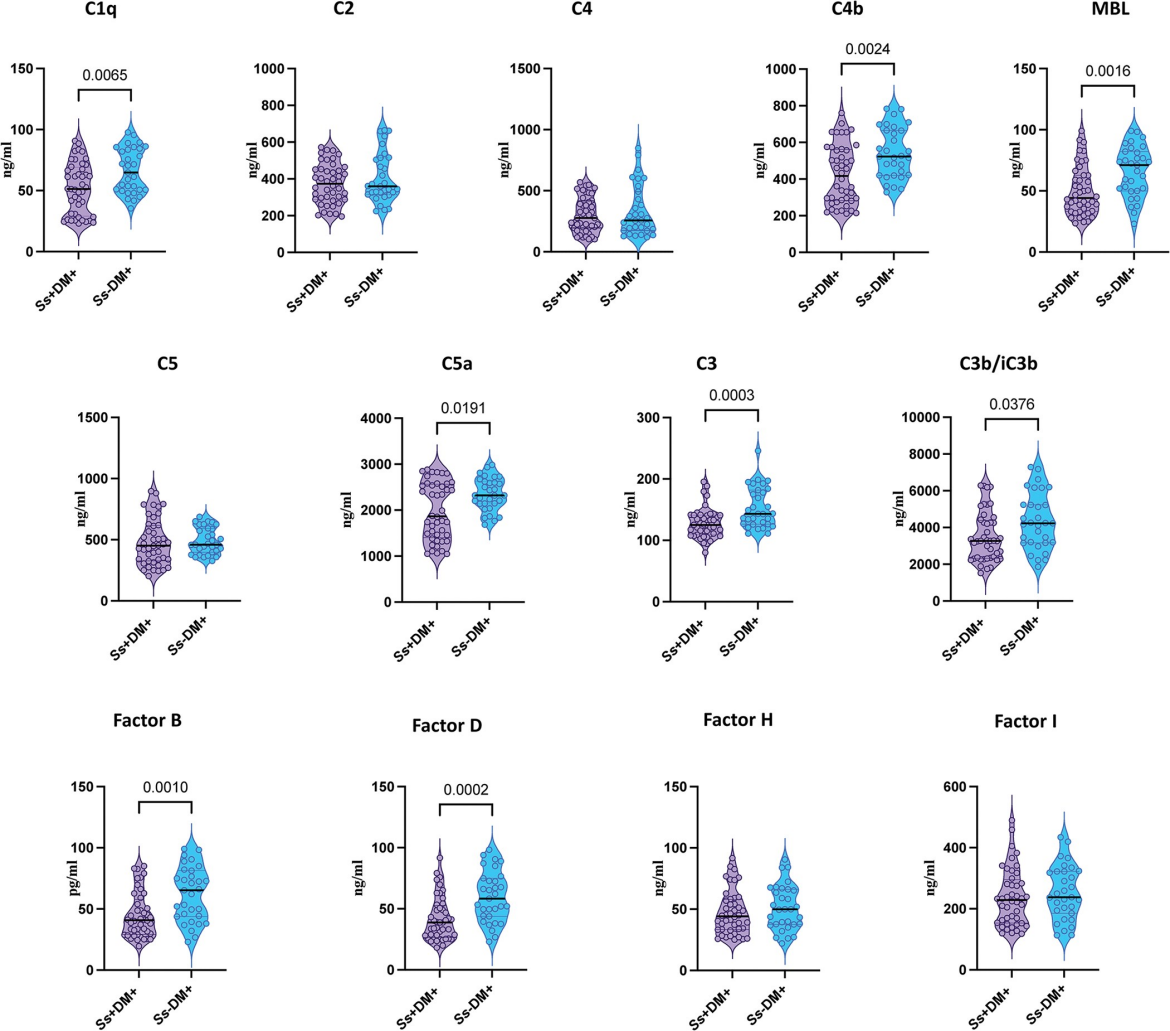
Diminished circulating levels of complement proteins and complement regulatory proteins in *Ss*+DM+ individuals. Plasma levels of complement proteins, C1q, C2, C3, C4, C4b, C5, C5a, MBL (Lectin), and complement regulatory proteins, Factor B, Factor D, Factor H, and Factor I in DM with *Ss*+ (n = 60) and without *Ss*- (n = 58) individuals. Each dot represents an individual subject and the bar represents the geometric mean (GM) and the dotted lines represent the upper and lower quartiles. Mann–Whitney U-test with Holms correction for multiple comparisons was done by p-value multiplied by the number of parameters.

To examine the impact of *Ss* infection on complement proteins and complement regulatory proteins in DM, we quantified the systemic levels of C1q, C2, C3, C4, C4b, C5, C5a, MBL (Lectin) complement proteins and complement regulatory proteins, Factor B, Factor D, Factor H and factor I in *Ss*+DM+ and *Ss*-DM+ individuals. As illustrated in Fig 1, complement proteins levels of C1q (Median [Mdn] of 51.36 ng/ml in *Ss*+DM+ compared to 64.84 ng/ml in *Ss*-DM+; p = 0.0065), C3 (Mdn of 125.1 ng/ml in *Ss*+DM+ compared to 143.1 ng/ml in *Ss*-DM+; p = 0.0003), C4b (Mdn of 416.8 ng/ml in *Ss*+DM+ compared to 522.7 ng/ml in *Ss*-DM+; p = 0.0024), C5a (Mdn of 1865 ng/ml in *Ss*+DM+ compared to 2322 ng/ml in *Ss*-DM+; p = 0.0191), MBL (Mdn of 44.14 ng/ml in *Ss*+DM+ compared to 71.00 ng/ml in *Ss*-DM+; p = 0.0016), Factor B (Mdn of 40.87 ng/ml in *Ss*+DM+ compared to 65.28 ng/ml in *Ss*-DM+; p = 0.0010), and Factor D (Mdn of 38.71 ng/ml in *Ss*+DM+ compared to 58.25 ng/ml in *Ss*-DM+; p = 0.0002) were significantly lower in *Ss+*DM+ compared to *Ss-* DM+ individuals. Thus, *Ss* infection is associated with decreased levels of complement proteins, C1q, C4b, MBL (Lectin), C3, C5a, and C3b/iC3b and complement regulatory proteins, Factor B and Factor D in individuals with DM.

### 3.3. Principal component analysis (PCA) reveals trends in complement proteins and complement regulatory proteins

**Fig 2:**
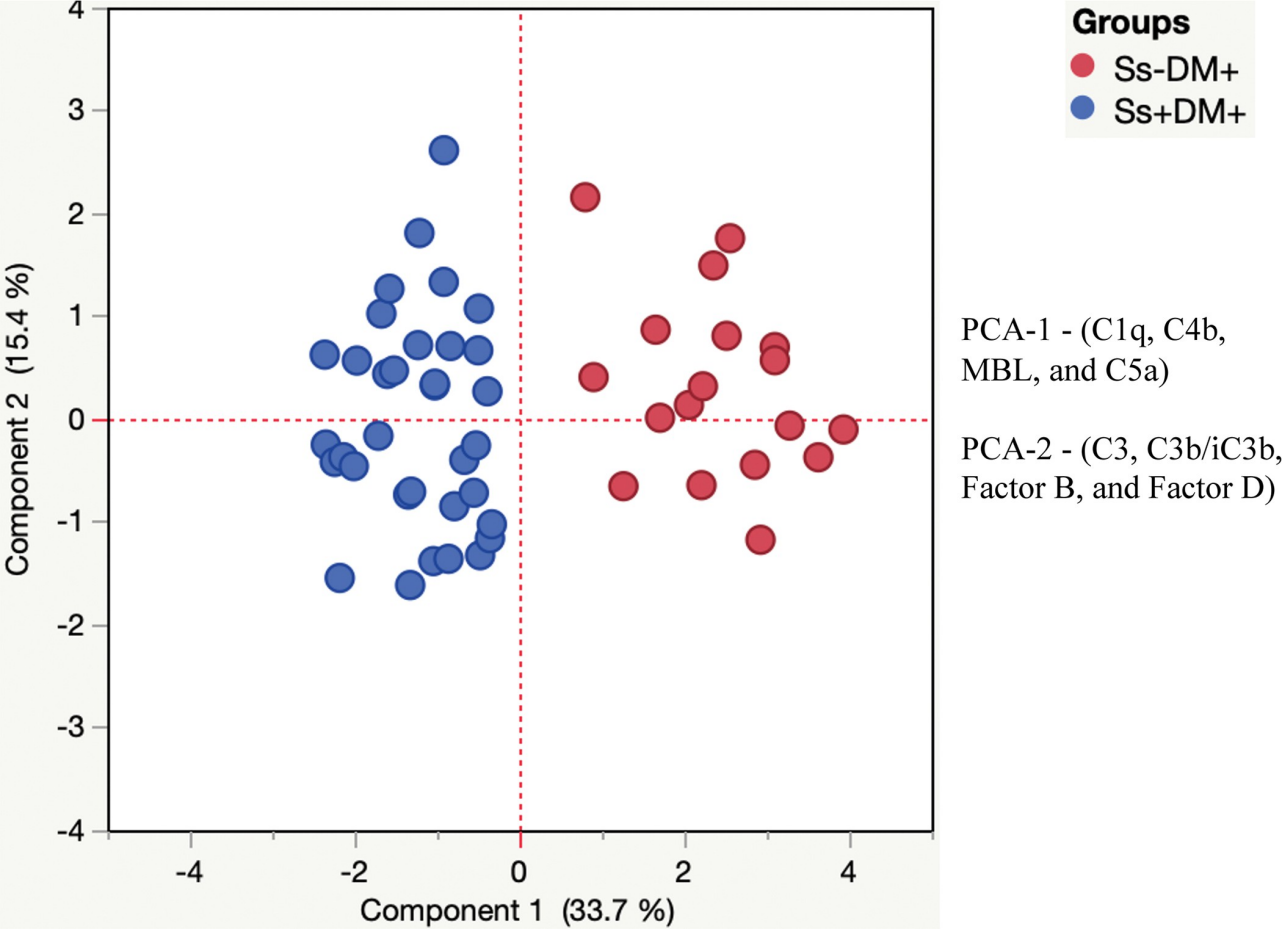
Principal component analysis reveals trends in complement proteins and complement regulatory proteins. Principal component analysis (PCA) was performed to show the distribution of data from the combination of three groups *Ss*+DM+ (blue circles), and *Ss*-DM+ (maroon circles). The PCA represents the two principal components of variation. PCA analysis was performed with complement proteins and complement regulatory proteins between *Ss*+ and *Ss*- individuals with DM.

To visualize the clustering pattern of complement proteins and complement regulatory proteins in DM individuals with or without *Ss* infection. we performed PCA with complement proteins and regulatory proteins (C1q, C2, C3, C4, C4b, C5, C5a, MBL, Factor B, Factor D, Factor H, and Factor I). After excluding those factors with commonalities as low as 0.5, we assessed PCA-1 (C1q, C4b, MBL, and C5a) and PCA-2 (C3, C3b/iC3b, Factor B, and Factor D). As illustrated in Fig 2, PCA analysis showed that complement protein and complement regulatory protein clusters varied between *Ss*+DM+ and *Ss*-DM+ individuals. The score plot of the first two components revealed 33.7% and 15.4% of overall variance, respectively.

### 3.4. Alterations in systemic levels of complement proteins following anthelmintic treatment

**Fig 3:**
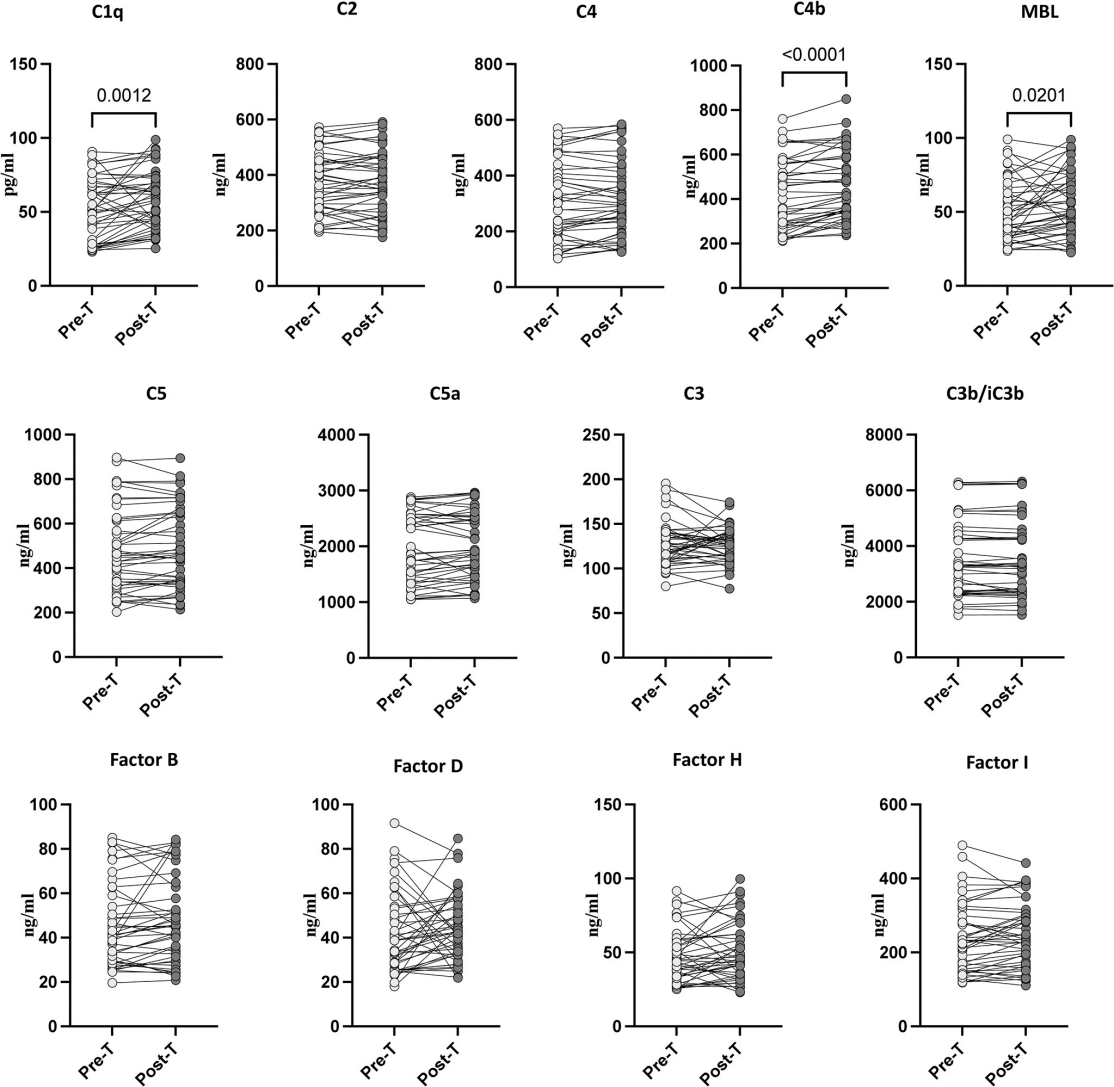
Alterations in systemic levels of complement proteins following anthelmintic treatment. Plasma levels of complement proteins, C1q, C2, C3, C4, C4b, C5, C5a, MBL, and complement regulatory proteins, Factor B, Factor D, Factor H, and Factor I in DM with *Ss*+ pre-treatment [Pre-T] [n = 60] and 6 months following treatment [post-T] [n = 60] were measured. p values were calculated using the Wilcoxon matched pair test.

To determine the effect of anthelmintic treatment in *Ss*+ DM+ individuals on the systemic levels of C1q, C2, C3, C4, C4b, C5, C5a, MBL (Lectin) complement proteins and complement regulatory proteins like Factor B, Factor D, Factor H, and Factor I. At 6 months following anthelmintic treatment, as shown in Fig 3, the levels of C1q (percentage increase of 15%; p = 0.0012), C4b (percentage increase of 11%; p<0.0001), and MBL (Lectin) (percentage increase of 11%; p = 0.0201) increased following treatment. Thus, anthelmintic treatment is associated with partial reversal of complement protein level alterations.

## 4. Discussion

Our study analysed the levels of complement proteins and complement regulatory proteins in DM with or without coincident *Ss* infection. We also examined the effect of anthelmintic treatment on these complement proteins and complement regulatory proteins. Emerging epidemiological and experimental studies have shown that helminth infections protect against the development and exacerbation of DM and other metabolic disorders in the host [5–8,14–17]. Recently, metabolic disorders with coincident helminth infections have gained attention owing to the suggestion that concomitant helminth infections may facilitate a beneficial impact on metabolic impairment [18,19]. Also, several reports determined that helminth infections have a beneficial effect on insulin resistance, dyslipidemia, and the pathology associated with DM [5,6,14,20–23]. Hence, it is important to understand the mechanism and interaction between helminths and metabolic disorders. In the current study, we have examined the complement activation pathways in DM individuals with or without *Ss* infection to understand this interaction.

Mounting evidence indicates that the complement system plays a key role in the pathogenesis of diabetes and its complications [24–28]. A recent study determined that increased levels of C3 were associated with a greater likelihood of diabetic complications [1–3]. Both human and animal studies determined that plasma levels of C3, C4, C4b, iC3b, MBL, MASP-2, C5b-9 Factor B, and Bb were found to be significantly increased in diabetes condition and have a role in exacerbating the pathophysiology of diabetes mellitus [1,25–27,29–31]. In earlier publications from our group, we have shown that DM individuals exhibited elevated levels of cytokines, chemokines and acute phase proteins when compared with DM and helminth individuals [5–7,13]. Our data here clearly shows that there is a link between lower levels of C1q, C4b, MBL, C5a, C3, C3b/iC3b factor B and factor D and the presence of *Ss* infection. The PCA results describe the complement protein and complement regulatory protein clusters that differ between *Ss*+DM+ and *Ss*-DM+ individuals. Following anthelmintic treatment, pro-inflammatory cytokines, chemokines and acute-phase proteins were reversed [5–7]. Similarly, following anthelmintic treatment, a substantial reversal of C1q, C4b and MBL parameters has been observed in our study. Similarly, we have previously shown (using the same patient cohort) that concomitant *Ss* infection exhibited diminished systemic levels of cytokine, chemokine, adipokine, and hormonal responses that favour protection from insulin resistance, pancreatic beta cell exhaustion, and angiogenic factors and soluble receptor for advanced glycation end product (RAGE) ligands and these levels were significantly reversed following anthelmintic treatment. Earlier studies have shown that complement activation is mediated by cytokines, and growth factors [32]. C3a and C5a increase T cell proliferation and cytokine release on APC, which promotes a shift towards Th1 immunity [1]. Our study findings indicate that in individuals with both helminth infection and diabetes mellitus (DM), helminths may influence the reduction of complement protein levels. This reduction potentially plays a role in mitigating inflammation and could potentially affect the severity of type 2 diabetes (T2D). However, it is important to note that while our study provides evidence of the impact on complement proteins, we currently lack direct evidence linking this alteration to changes in the severity of T2D.

Several studies have assessed the clinical and epidemiological relationship between helminth infection and metabolic disorders, obesity, and DM, but only a small number of studies have examined the underlying mechanism behind this interface. Our study has certain limitations in that it is not a randomized controlled trial; hence, the impact of elevated complement and complement proteins following the anthelmintic therapy may be due to either suppression of *Ss* infection or the anthelmintic medications (ivermectin and albendazole) or both. Also, we have not measured insulin resistance. We have not studied the effect of antidiabetic drugs on the post-treatment samples. Our data suggests that Ss infection lowers levels of various complement proteins, potentially impacting the severity of T2D, although formal demonstration of this effect on T2D severity remains pending.

Taken together, our data illustrate the positive impact of helminth infections on DM individuals concerning complement proteins and complement regulatory proteins dysregulation and suggest potentially more precise treatment approaches that might be used as adjunctive therapy for DM. This might be beneficial for the prevention of some of the complications of DM.
